# Supplementary material for: Expression of Checkpoint Molecules in the Tumor Microenvironment of Intrahepatic Cholangiocarcinoma: Implications for Immune Checkpoint Blockade Therapy
Source: Cells. 2023 Mar 9;12(6):851. doi: 10.3390/cells12060851 (PMC10047585; doi:10.3390/cells12060851)
Supplement: Supplementary file 1 [file cells-12-00851-s001.zip › cells-2209758-supplementary.pdf]

Supplementary Information

Supplementary Table S1. Antibodies and

Antibody	Marker	Dilution	Incubation time	Manufacturer
**CD4**	T cell	1:500	30 min	Dako
**CD8**	T cell	1:500	30min	Dako
**CD68**	Macrophages	1:6000	30 min	Dako
**TIM-3**	Checkpoint	1:500	Over night	Abcam
**TIGIT**	Checkpoint	1:200	Over night	Abcam
**LAG-3/CD223**	Checkpoint	1:300	Over night	LSBIO
**ICOS/CD278**	Checkpoint	1:200	Over night	Abcam
**CTLA-4/CD152**	Checkpoint	1:300	Over night	Origene
**PD-1/CD279**	Checkpoint	1:250	Over night	Abcam
**PD-L1/CD274**	Checkpoint	1:200	Over night	Dako
**PD-L2/CD273**	Checkpoint	1:400	Over night	Abcam

Supplementary Table S2 ICCA p-values

	Tumor - Sclerotic tumor	Tumor - Interface	Tumor - Normal	Interface - Normal	Interface - Sclerotic tumor	Normal - Sclerotic tumor
**CD4**	0.9854	0.1334	**0.034**	0.5306	0.1081	**0.0246**
**CD4_CTLA**	0.6117	0.1559	0.115	0.7733	**0.02**	**0.0125**
**CD4_ICOS**	0.0975	0.278	0.2021	0.8192	0.5014	0.6635
**CD4_ICOS_CTLA**	0.6935	0.4655	0.5132	0.9454	0.3888	0.4069
**CD4_ICOS_Tigit**	0.4674	0.9205	0.4242	0.634	0.4641	0.2523
**CD4_ICOS_Tigit_CTLA**	0.2956	0.7986	0.6369	0.6137	0.4054	0.2396
**CD4_Lag3**	nan	0.1482	0.7459	0.4517	nan	nan
**CD4_Lag3_Tim3**	nan	0.3761	0.9672	0.6586	nan	nan
**CD4_PD1**	nan	0.073	0.3317	0.5908	nan	nan
**CD4_PD1_Lag3**	nan	0.1783	0.6057	0.6142	nan	nan
**CD4_PD1_Lag3_Tim3**	nan	0.3514	0.9856	0.6218	nan	nan
**CD4_PD1_Tim3**	nan	**0.0278**	0.4509	0.4072	nan	nan
**CD4_Tigit**	0.1067	0.9408	0.6009	0.6855	0.2075	0.0588
**CD4_Tigit_CTLA**	0.1471	0.8192	0.5588	0.5842	0.2778	0.0892
**CD4_Tim3**	nan	**0.0137**	0.3329	0.4966	nan	nan
**CD68**	0.1437	0.1072	0.4734	0.818	0.7282	0.7421
**CD68_PD1**	0.2098	0.7637	0.8833	0.8797	0.1436	0.2132
**CD68_PD1_PDL1**	0.4912	0.7159	0.4846	0.6579	0.406	0.3386
**CD68_PD1_PDL1_PDL2**	0.3972	0.7537	0.5039	0.6588	0.3911	0.3311
**CD68_PD1_PDL2**	0.4561	0.7906	0.5152	0.6545	0.4621	0.3557
**CD68_PDL1**	0.9133	0.8463	0.8408	0.679	0.871	0.7271
**CD68_PDL1_PDL2**	0.3917	0.6654	0.922	0.6077	0.4693	0.37
**CD68_PDL2**	0.2882	0.8642	0.8881	0.7701	0.4446	0.2828
**CD8**	**0.0002**	**0.0**	**0.0002**	**0.0169**	**0.0029**	0.5177
**CD8_CTLA**	0.5446	0.2597	0.8321	0.5363	0.1134	0.4819
**CD8_ICOS**	0.1359	0.3099	0.4504	0.2544	**0.0309**	0.8822
**CD8_ICOS_CTLA**	0.2274	0.384	0.5009	0.4016	0.1442	0.9009
**CD8_ICOS_Tigit**	0.4267	0.2713	0.8943	0.4729	0.2079	0.5621
**CD8_ICOS_Tigit_CTLA**	0.2321	0.6566	0.4891	0.4251	0.2031	0.5882
**CD8_Lag3**	nan	0.5195	0.2751	0.8118	nan	nan
**CD8_Lag3_Tim3**	nan	0.6208	0.9332	0.713	nan	nan
**CD8_PD1**	0.4439	0.6546	0.8904	0.8548	0.6859	0.6274
**CD8_PD1_Lag3**	nan	0.0801	0.2062	0.6482	nan	nan
**CD8_PD1_Lag3_Tim3**	nan	0.2781	0.6883	0.5197	nan	nan
**CD8_PD1_PDL1**	0.2249	0.1632	0.4447	0.2718	**0.0288**	0.7746
**CD8_PD1_PDL1_PDL2**	0.4512	0.0534	0.3639	0.2618	0.0675	0.4678
**CD8_PD1_PDL2**	0.408	**0.0411**	0.3806	0.2443	0.0661	0.548
**CD8_PD1_Tim3**	nan	0.2145	0.648	0.327	nan	nan
**CD8_PDL1**	0.8644	0.142	0.6785	0.4773	**0.0112**	0.5338
**CD8_PDL1_PDL2**	0.5907	0.2071	0.9464	0.3046	0.0792	0.6236
**CD8_PDL2**	0.3476	0.7645	0.8704	0.6818	0.6523	0.3619
**CD8_Tigit**	0.7365	0.4061	0.7947	0.7311	0.2109	0.5941
**CD8_Tigit_CTLA**	0.5454	0.4608	0.6682	0.3784	0.2021	0.978
**CD8_Tim3**	nan	0.7116	0.9485	0.8235	nan	nan
